# A 98 years old super elderly coronary heart disease patient treated with enhanced external counterpulsation: a case report

**DOI:** 10.3389/fcvm.2025.1616457

**Published:** 2025-10-31

**Authors:** Cailong Lin, Yingying Lai, Jiajia Xu, Min Li, Yan Guo, Jianyu Huang, Lin Xu

**Affiliations:** ^1^ Department of Geriatric Cardiology, General Hospital of Southern Theater Command, Guangzhou, China; ^2^ Guangdong Pharmaceutical University, Guangzhou, China

**Keywords:** enhanced external counterpulsation, coronary heart disease, super elderly, case report, clinical efficacy

## Abstract

Enhanced external counterpulsation (EECP) is a safe, non-invasive, economic and effective assisted circulation technique. It not only can improves multisystem disorders, but also reduces adverse event rates. EECP utilizes electrocardiogram-gated sequential inflation of cuffs wrapped around patient's lower extremities to increase blood flow perfusion of heart, which can improve myocardial ischemia of patients with coronary heart disease (CHD). Patients with CHD or heart failure or sleep disorder are suitable to receive EECP. But there are few reports on super elderly people. This is the first reported case of a 98-year-old patient with CHD, who accepted EECP. Patient was presented to hospital with complaints of “intermittent chest tightness for 12 years, worsening for 20 days.” His medical history included lacunar infarction, as well as type 2 diabetes mellitus and hypertension. For the elderly patients reported in this case, the efficacy of medicine was limited and the surgical risk was high. After performing a course of EECP, The patient's symptoms of CHD adverse psychological conditions and sleep quality were improved. This report proved the effectiveness and safety of EECP to treat such super elderly patients with CHD, which provided evidence for the clinical application of EECP in such patients.

## 1 Introduction

At present, the main treatment methods for coronary heart disease (CHD) are medicine and percutaneous coronary intervention (PCI), but for super elderly patients, the efficacy of medicine is limited and the surgical risk is high. Even when patients with coronary artery disease (CAD) are treated with antianginal medications, and/or undergo procedures like PCI or coronary artery bypass grafting, the percentage of those experiencing daily or weekly angina still between 2% and 24% (1). The European Society of Cardiology Guidelines consider EECP to be one of the two most promising and easy to implement in daily clinical work among existing treatment options (2). Enhanced external counterpulsation (EECP) is a safe, non-invasive, economic and effective assisted circulation technique. It not only can improves multisystem disorders, but also reduces adverse event rates. EECP utilizes electrocardiogram-gated sequential inflation of cuffs wrapped around patient's lower extremities to increase blood flow perfusion of heart, which can improve myocardial ischemia of patients with CHD (3). Therefore, EECP is recommended in treatment or management guidelines (3–5), but there are few reports on super elderly people. Here, we reported the case of a 98-year-old patient with CHD who accepted EECP. No prior reports described patients of such an advanced age receiving EECP before. The safety and efficacy of EECP in this population remains unknown. This report provided evidence for the clinical application of EECP in super elderly CHD patient. Informed consent was obtained from the patient for publication of this case report details.

## 2 Case description

### 2.1 History and physical exam

The patient is a 98-year-old male, and was admitted to our hospital due to intermittent chest tightness for 12 years, worsening for 20 days. He had intermittent chest tightness in the precordial area without obvious cause since 2011. Each time lasted about 5 min, which relieved after rest. He was diagnosed with coronary atherosclerotic heart disease and was discharged after accepting medicines to relieve symptoms. The above symptoms recurred intermittently therefore. But 20 days before admission, the symptoms occurred more frequently and seriously than before, 4–5 times a day. Each episode lasted about 10 min and required medication or prolonged rest for relief. The patient had some past history of CHD, lacunar infarction, type 2 diabetes mellitus, hypertension. He had no history of genetic diseases, and no symptoms of anxiety or depression after taking an anxiety and depression scale assessment. The physical examinations were as follow: body temperature, 36.6℃; pulse, 80 times/min; respiratory rate, 20 times/min; blood pressure, 130/70 mmHg.

### 2.2 Condition analysis

The patient continued to receive medications such as anticoagulant, antihypertensives, lipid-lowering agents, gastroprotective agents, vasodilator and others. But the symptoms had not been fully relieved. Elderly patients with CHD usually have higher coronary calcium scores, which can mask the true degree of vascular stenosis (6). The microvascular disease caused by diabetes may lead to the decrease of glomerular filtration rate (7). Coronary computed tomography angiography (CCTA) needs to inject iodine contrast agent. The elderly CHD patients, especially those with diabetes, are at high risk of contrast-induced nephropathy (8). Therefore, we did not choose CCTA. We wanted to perform coronary angiography for him, however, his family members refused after being informed the relevant risks. Because the patient was very elderly and they are afraid that he may not be able to withstand the surgery. To reduce the frequency of chest tightness and lower the risk of cardiovascular events, we proposed cardiac rehabilitation. EECP has the effects of relieving symptoms of CHD, improving cardiac function, reducing the area of myocardial ischemia, optimizing medication, and improving quality of life. The patient had been diagnosed with CHD, which is a well-recognized indication for EECP. And there were no contraindications found in the safety assessment, which included basic and specialized examinations mentioned in the expert consensus (3). Blood routine, coagulation function, blood lipids, blood glucose, liver and kidney function, routine electrocardiogram, cardiac color ultrasound, lower limb vascular ultrasound were basic assessment examinations. Dynamic electrocardiogram, dynamic blood pressure, and non-invasive hemodynamic examinations are specialized evaluation examinations. After obtaining the consent of the patient and his family members, we decided to give him a course of EECP.

### 2.3 Protocol

**Figure 1:**
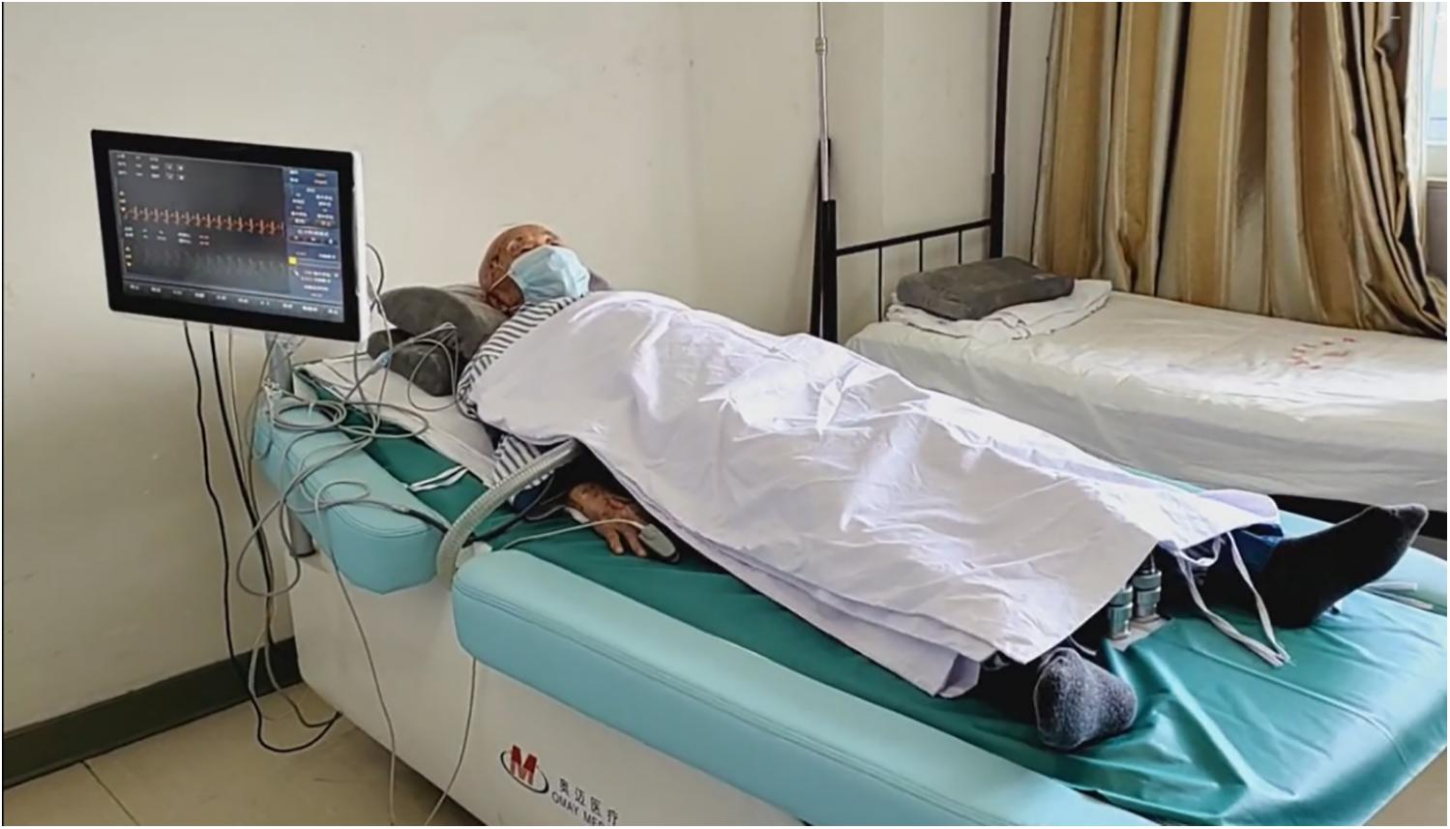
Patient's vital signs were monitored during enhanced external counterpulsation.

The pressure was set to 0.013 MPa. EECP sessions were administered for a total of 35 h over seven weeks (5 days per week), with each daily session lasting 1 h (divided into two 0.5-hour segments: morning and afternoon). The patient's vital signs, including blood pressure, heart rate, and oxygen saturation, were continuously monitored during each EECP session (Figure 1).

## 3 Results

**Figure 2:**
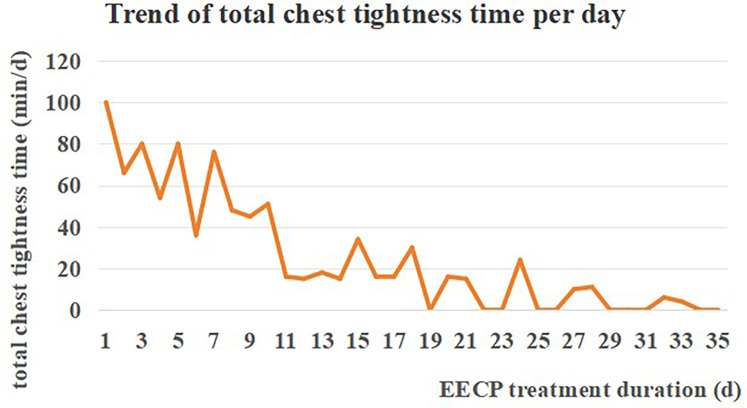
The patient's symptom of chest tightness was improved.

Following drugs and EECP, the patient indicated that the symptom of chest tightness was improved, and his total duration of chest tightness decreased from a maximum of 100 min/d to a minimum of 0 min/d (Figure 2). At admission, the patient was unable to ambulate independently. However, after completing the EECP course, he could walk 5 meters with railing support or assistance from others. Both the patient and his family were satisfied with the recovery results and expressed their commitment to continue with EECP.

The primary clinical evaluations before and after EECP are shown in Table 1. Regarding cardiac function indicators, results exhibited a decrease in systolic blood pressure (SBP), while diastolic blood pressure (DBP) did not change significantly. Ejection fraction (EF) and fractional shortening (FS) remained stable, whereas cardiac output, cardiac index and other indicators demonstrated an increase. In terms of clinical biochemical results, blood lipids and electrolyte metabolism were improved, urinary bacteria and urinary microalbumin (UmALB) levels were reduced, myocardial enzyme indicators were not significantly abnormal. Regarding mental health assessment scale, scores on anxiety, depression and sleep scales showed slight but clinically meaningful improvements.

**Table 1:** Clinical evaluation before and after enhanced external counterpulsation.

Clinical evaluation	Before	After
Blood pressure		
SBP (mmHg)	139	131
DBP (mmHg)	69	68
Echocardiography		
EF (%)	61	63
FS (%)	33	34
Non-invasive Hemodynamic examinations		
Cardiac output (L/min)	1.7	4.8
Cardiac index (L/min·㎡)	1.1	3
Stroke volume (mL/beat)	22.5	82.1
Stroke index (mL/beat·㎡)	14	51
Blood lipids		
Triglyceride (mmol/L)	0.74	0.51
Total cholesterol (mmol/L)	3.31	3.21
LDL-C (mmol/L)	1.5	1.26
HDL-C (mmol/L)	1.51	1.59
Apolipoprotein A-Ⅰ (g/L)	1.16	1.05
Apolipoprotein B (g/L)	0.62	0.51
Lipoprotein (a) (g/L)	0.35	0.23
Serum electrolytes		
Mg (mmol/L)	0.66	0.82
K (mmol/L)	2.90	3.50
Ca (mmol/L)	2.06	2.40
*P* (mmol/L)	0.88	0.96
Urine routine and metabolic panel		
Bacteria (/μl)	6,315	12
Red blood cell (/μl)	21	3.4
UACR (mg/gCr)	34.5	29.1
UmALB (mg/L)	20.6	12.2
Myocardial enzyme		
CK (U/L)	47	49
CK-MB (U/L)	31	39
LD (U/L)	34	35
α-HBDH (U/L)	132	120
Hospital anxiety and depression scale		
Anxiety score	2	1
Depression score	2	1
Pittsburgh sleep quality index		
Sleep score	4	3

SBP, systolic blood pressure; DBP, Diastolic blood pressure; EF, Left ventricular ejection fraction; FS, Left Ventricular Fraction Shortening; LDL, Low-density lipoprotein cholesterol; HDL-C, High-density lipoprotein cholesterol; Mg, Magnesium; K, Potassium; Ca, Calcium; P, Inorganic phosphate; UACR, Urine albumin creatine ratio; UmALB, Urinary microalbumin; CK, Creatine kinase; CK-MB, Creatine kinase-MB; LD, Lactate dehydrogenase; α-HBDH, α-hydroxybutyric dehydrogenase.

After 6 months of follow-up, the patient had no major adverse cardiovascular events. Based on the comprehensive comparison of pre- and post-EECP, the case showed that EECP was both effective and safe for super elderly patients with CHD.

## 4 Discussion

In terms of the therapeutic effect, EECP could not only effectively reduce the frequency and shorten the duration of chest tightness, but also increase the patient's walking distance. It might be related to the positive effects of EECP on coronary perfusion and flow (1, 4–7). The study carried out by Michel et al. (9) revealed that EECP has the ability to boost coronary artery blood flow velocity and pressure, and also optimize left ventricular hemodynamics. In Urano's study (10), EECP could improve exercise duration and tolerance of CAD patients.

**Figure 3:**
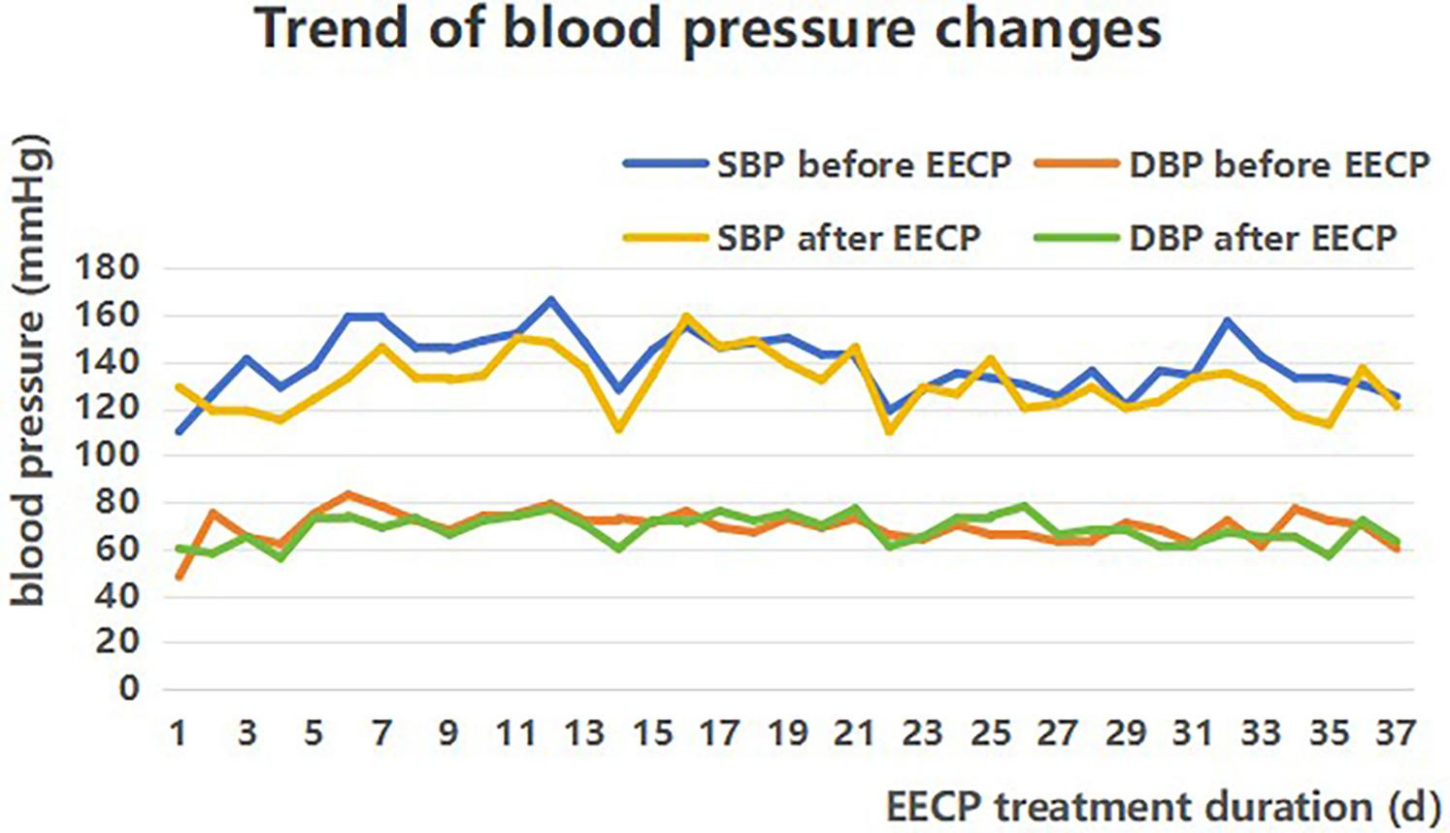
Blood pressure's changes of patient during enhanced external counterpulsation.

The reduction of SBP which might be due to EECP's ability to reduce afterload and total peripheral resistance (11). But the effect on DBP was not significant (Figure 3). Our case was similar to that in Alex's research (12), which demonstrated that EECP can increase SBP in patients without significantly affecting DBP or HR. The change in DBP is not significant, which may be related to the physical characteristics of EECP that mainly increases cardiac blood flow perfusion during diastole, in order to avoid increasing cardiac afterload on systole.

Besides, the EF and FS remain basically unchanged, possibly because of the reduced counterpulsation pressure. Although EECP can improve coronary blood flow and increase oxygen supply, which is beneficial for heart function (3), the “afterload reduction” effect caused by EECP may mask the detection of substantial improvement in myocardial contractility in the short term. As a result, a longer treatment duration is required for EF and FS to exhibit significant changes, especially in patients with poor baseline conditions or among the super elderly.

Due to the patient's advanced age and limited mobility, he couldn't able to meet the core requirements of the 6-minute walking test and cardiopulmonary exercise test. If the patient forcefully attempts, it may lead to falls, fractures, or cardiovascular events. Non-invasive hemodynamic monitoring (NHM) provides hemodynamic data without requiring exercise, thus avoiding exercise risks (13). It is suitable for bedridden or weak patients or those with contraindications to exercise. NHM was therefore used to evaluate cardiopulmonary function in this patient. It was found that the patient's cardiac output, cardiac index, and other indicators had increased after one course of treatment, which indicating the decrease in cardiac preload and afterload resistance, and a recovery in myocardial contractility (14, 15).

Meanwhile, the patient's blood lipids were regulated, the exchange and metabolism of blood electrolytes were increased, and the number of urinary bacteria and urinary microalbumin were decreased. Research on EECP mainly focuses on the mechanisms of improving angina, cardiac function, or endothelial function, while few authoritative studies directly address lipid-lowering or promoting metabolic exchange and metabolism. However, EECP can increase blood perfusion to important organs, improve endothelial function, promote angiogenesis and may exert potential effects on regulating blood lipid levels and increasing substance exchange (3). In this case, we observed that EECP enhanced the frequency of urination in patients, consistent with precautions described in multiple guidelines (2–5). We hypothesized that increasing urinary output frequency may effectively flush the urinary system, thereby reducing the number of urinary bacteria and urinary microalbumin.

From the safety perspective, all the other myocardial enzyme values fluctuated within the normal range except for a minor decrease in *α*-HBDH, and no adverse cardiovascular events occurred in the patient after 6 months of treatment. In addition, the decrease in anxiety and depression scores and Pittsburgh Sleep Scale score manifested that patient's quality of life has improved. It may be related to EECP reduced stress and daytime dysfunction, improved mood and sleep quality (16, 17). Xiuli Xu's research found that EECP can improve the sleep quality and psychological quality of life of patients with chronic insomnia (16). Additionally, the patient's progression from bedridden status to ambulation with assistance may be attributed to enhanced exercise tolerance secondary to EECP therapy. It was similar to the research of Huongrui Yang and LingZhong (15, 18).

Based on the special characteristics of the super elderly, there are two precautions proposed. Firstly, develop individualized treatment protocol based on the patient's condition. The parameter of this case at the beginning was set as 0.020 MPa, but the patient complained of pain during EECP (1). Considering that the patient is 98 years old with multiple basic diseases, and less subcutaneous adipose and muscle, the parameters are set to 0.013 MPa after repeated adjustments and trials. Although the parameters are below the guideline range, good therapeutic effects can still be achieved. Secondly, evaluation before EECP and safety monitoring during the process. A series of security assessment need to be performed before EECP to ensure that patients have no contraindications.

To our knowledge, this is the first report of a case of a 98-year-old CHD patient who accepted EECP. This case indicates that EECP can both improve symptoms of CHD and reduce the incidence of adverse cardiovascular events while alleviating adverse psychological conditions to improve the quality of life. The case provides ideas and references for the treatment of similar cases, and provides evidence for the clinical application of EECP in super elderly coronary heart disease. The limitation is that the patient didn't performer the 6-minute walking test and cardiopulmonary exercise test to evaluate cardiopulmonary function.

For 98-year-old elderly patients undergoing EECP, there are concerns about physiological vulnerability, risk of complications, and tolerance issues. Buschman et al. proposed individual shear rate therapy for lower extremity peripheral artery disease based on EECP (19). Compared with EECP, ISRT significantly enhanced the tolerability, safety and efficacy of LEAD patients (20, 21). To ensure that patient can do cardiac rehabilitation safely, we will consider performing ISRT for him in the future.

## Data Availability

The datasets presented in this article are not readily available due to the protection of patient privacy. Requests to access the datasets should be directed to the corresponding author.

## References

[1] Davies A Fox K Galassi AR Banai S Ylä-Herttuala S Lüscher TF. Management of refractory angina: an update. Eur Heart J. (2021) 42(3):269–83. 10.1093/eurheartj/ehaa820 33367764

[2] Vrints C Andreotti F Koskinas KC Rossello X Adamo M Ainslie J 2024 ESC guidelines for the management of chronic coronary syndromes. Eur Heart J. (2024) 45(36):3415–537. 10.1093/eurheartj/ehae177 39210710

[3] Cardiovascular Group, Geriatrics Branch, Chinese Medical Association, Editorial Board of the Chinese Journal of Geriatrics, Gerontology Group, External Counterpulsating Branch, Chinese Society of Biomedical Engineering. Expert consensus on the clinical application of enhanced external counterpulsation in elderly people. Chinese Journal of Geriatrics. (2019) 38(9):953–61. 10.3760/cma.j.issn.0254-9026.2019.09.001

[4] Lin S Xiao-Ming W Gui-Fu W. Expert consensus on the clinical application of enhanced external counterpulsation in elderly people (2019). Aging Medicine (Milton). (2020) 3(1):16–24. 10.1002/agm2.12097 PMC7099759 32232188

[5] Virani SS Newby LK Arnold SV Bittner V Brewer LC Demeter SH 2023 AHA/ACC/ACCP/ASPC/NLA/PCNA guideline for the management of patients with chronic coronary disease: a report of the American Heart Association/American College of Cardiology joint committee on clinical practice guidelines. Circulation. (2023) 148(13):e148. 10.1161/CIR.0000000000001183 37471501

[6] Mortensen MB Gaur S Frimmer A Bøtker HE Sørensen HT Kragholm KH Association of age with the diagnostic value of coronary artery calcium score for ruling out coronary stenosis in symptomatic patients. JAMA Cardiol. (2022) 7(1):36–44. 10.1001/jamacardio.2021.4406 34705022 PMC8552116

[7] DeFronzo RA Reeves WB Awad AS. Pathophysiology of diabetic kidney disease: impact of SGLT2 inhibitors. Nat Rev Nephrol. (2021) 17(5):319–34. 10.1038/s41581-021-00393-8 33547417

[8] Silver SA Shah PM Chertow GM Harel S Wald R Harel Z. Risk prediction models for contrast induced nephropathy: systematic review. Br Med J. (2015) 351:h4395. 10.1136/bmj.h4395 26316642 PMC4784870

[9] Michaels AD Accad M Ports TA Grossman W. Left ventricular systolic unloading and augmentation of intracoronary pressure and Doppler flow during enhanced external counterpulsation. Circulation. (2002) 106(10):1237–42. 10.1161/01.cir.0000028336.95629.b0 12208799

[10] Urano H Ikeda H Ueno T Matsumoto T Murohara T Imaizumi T. Enhanced external counterpulsation improves exercise tolerance, reduces exercise-induced myocardial ischemia and improves left ventricular diastolic filling in patients with coronary artery disease. J Am Coll Cardiol. (2001) 37(1):93–9. 10.1016/s0735-1097(00)01095-0 11153780

[11] Zhang DD Wang SH Ma J Zhao SH Chen XM Sun YK Immediate and lasting effects of enhanced external counterpulsation on blood pressure in elderly patients with hypertension. Chin J Geriatr. (2021) 40(12):1512–6. 10.3760/cma.j.issn.0254-9026.2021.12.008

[12] Campbell AR Satran D Zenovich AG Campbell KM Espel JC Arndt TL Enhanced external counterpulsation improves systolic blood pressure in patients with refractory angina. Am Heart J. (2008) 156(6):1217–22. 10.1016/j.ahj.2008.07.024 19033023

[13] Sirkiä JP Panula T Kaisti M. Non-Invasive hemodynamic monitoring system integrating spectrometry, photoplethysmography, and arterial pressure measurement capabilities. Adv Sci (Weinh). (2024) 11(24):e2310022. 10.1002/advs.202310022 38647403 PMC11199981

[14] Guo WJ Li MM Zheng CP Ren XX Chen K Peng XY Effect of EECP on vascular endothelial growth factor and cardiopulmonary function in patients with SAP. Chin J Geriatr Heart Brain Vessel Dis. (2024) 26(12):1428–32. 10.3969/j.issn.1009-0126.2024.12.010

[15] Zhong L Xing J Zhao BL Wang CH. Effects of enhanced external counterpulsation combined with moderate-intensity interval training on cardiopulmonary reserve capacity and exercise endurance in coronary artery disease. Chin J Rehab Med. (2023) 38(4):478–84. 10.3969/j.issn.1001-1242.2023.04.007

[16] Xu X Zhou W Wang Y Wang Z Zhang X Zhang X Enhanced external counterpulsation improves sleep quality in chronic insomnia: a pilot randomized controlled study. J Affect Disord. (2024) 350:608–17. 10.1016/j.jad.2024.01.090 38218261

[17] Shi Y Li FJ. Effects of enhanced external counterpulsation combined with aerobic exercise on exercise tolerance and anxiety-depression in patients with coronary artery disease. J Hebei North Univ. (2022) 38(12):13–6. 10.3969/j.issn.1673-1492.2022.12.003

[18] Yang H Song L Ning X Ma Y Xue A Zhao H Enhanced external counterpulsation ameliorates endothelial dysfunction and elevates exercise tolerance in patients with coronary artery disease. Front Cardiovasc Med. (2022) 9:997109. 10.3389/fcvm.2022.997109 36523357 PMC9744945

[19] Buschmann EE Brix M Li L Doreen J Zietzer A Li M Adaptation of external counterpulsation based on individual shear rate therapy improves endothelial function and claudication distance in peripheral artery disease. Vasa. (2016) 45(4):317–24. 10.1024/0301-1526/a000544 27428501

[20] Soubh N Hillmeister P Buschmann E Klaproth C Buschmann I. Tolerability safety and effectiveness of enhanced external counterpulsation versus individual shear rate therapy in patients with lower extremity atherosclerotic disease: a prospective pilot clinical trial. Acta Physiol (Oxf). (2023) 237(3):e13913. 10.1111/apha.13913 36599365

[21] Picard F Panagiotidou P Wolf-Pütz A Buschmann I Buschmann E Steffen M Individual shear rate therapy (ISRT)-further development of external counterpulsation for decreasing blood pressure in patients with symptomatic coronary artery disease (CAD). Hypertens Res. (2020) 43(3):186–96. 10.1038/s41440-019-0380-x 31866668

