# Supplementary material for: A 98 years old super elderly coronary heart disease patient treated with enhanced external counterpulsation: a case report
Source: Front Cardiovasc Med. 2025 Oct 31;12:1616457. doi: 10.3389/fcvm.2025.1616457 (PMC12615481; doi:10.3389/fcvm.2025.1616457)
Supplement: Supplementary file 1 [file Datasheet1.pdf]

CARE Checklist of information to include when writing a case report

CARE case report guidelines

(cc) BY-NC-ND

Topic	Item	Checklist item description	Reported on Line
Title	1	The diagnosis or intervention of primary focus followed by the words "case report"	Title
Key Words	2	2 to 5 key words that identify diagnoses or interventions in this case report, including "case report"	Key words
Abstract (no references)	3a	Introduction: What is unique about this case and what does it add to the scientific literature?	Introduction
	3b	Main symptoms and/or important clinical findings	History and Physical Exam
	3c	The main diagnoses, therapeutic interventions, and outcomes	Condition Analysis
	3d	Conclusion—What is the main "take-away" lesson(s) from this case?	Discussion
Introduction	4	One or two paragraphs summarizing why this case is unique (**may include references**)	Discussion
Patient Information	5a	De-identified patient specific information.	Introduction
	5b	Primary concerns and symptoms of the patient.	History and Physical Exam
	5c	Medical, family, and psycho-social history including relevant genetic information	History and Physical Exam
	5d	Relevant past interventions with outcomes	History and Physical Exam
Clinical Findings	6	Describe significant physical examination (PE) and important clinical findings.	Results
Timeline	7	Historical and current information from this episode of care organized as a timeline	Results
Diagnostic Assessment	8a	Diagnostic testing (such as PE, laboratory testing, imaging, surveys).	Results
	8b	Diagnostic challenges (such as access to testing, financial, or cultural)	Discussion
	8c	Diagnosis (including other diagnoses considered)	History and Physical Exam
	8d	Prognosis (such as staging in oncology) where applicable	Results
Therapeutic Intervention	9a	Types of therapeutic intervention (such as pharmacologic, surgical, preventive, self-care)	Condition Analysis
	9b	Administration of therapeutic intervention (such as dosage, strength, duration)	Protocol
	9c	Changes in therapeutic intervention (with rationale)	Discussion
Follow-up and Outcomes	10a	Clinician and patient-assessed outcomes (if available)	Table 1
	10b	Important follow-up diagnostic and other test results	Discussion
	10c	Intervention adherence and tolerability (How was this assessed?)	Discussion
	10d	Adverse and unanticipated events	Discussion
Discussion	11a	A scientific discussion of the strengths AND limitations associated with this case report	Discussion
	11b	Discussion of the relevant medical literature **with references**.	Discussion
	11c	The scientific rationale for any conclusions (including assessment of possible causes)	Discussion
	11d	The primary "take-away" lessons of this case report (without references) in a one paragraph conclusion	Discussion
Patient Perspective	12	The patient should share their perspective in one to two paragraphs on the treatment(s) they received	Results
Informed Consent	13	Did the patient give informed consent? Please provide if requested	Yes ☑ No ☐
